# The Effect of Rhenium Content on Microstructural Changes and Irradiated Hardening in W-Re Alloy under High-Dose Ion Irradiation

**DOI:** 10.3390/nano13030497

**Published:** 2023-01-26

**Authors:** Fengfeng Luo, Hongtai Luo, Qiuxiang Liu, Liang Zhou, Wenbin Lin, Ziyang Xie, Liping Guo

**Affiliations:** 1 Software Engineering Institute of Guangzhou, Guangzhou 510990, China; 2 Key Laboratory of Artificial Micro- and Nano-Structures of Ministry of Education, Hubei Nuclear Solid Physics Key Laboratory, School of Physics and Technology, Wuhan University, Wuhan 430072, China; 3 Institute of Applied Physics, Jiangxi Academy of Science, Nanchang 330029, China

**Keywords:** W-Re alloy, high-dose irradiation, dislocation loops, voids, irradiated hardening

## Abstract

An amount of 100 dpa Si^2+^ irradiation was used to study the effect of transmutation rhenium content on irradiated microscopic defects and hardening in W-xRe (x = 0, 1, 3, 5 and 10 wt.%) alloys at 550 °C. The increase in Re content could significantly refine the grain in the W-xRe alloys, and no obvious surface topography change could be found after high-dose irradiation via the scanning electron microscope (SEM). The micro defects induced by high-dose irradiation in W and W-3Re alloys were observed using a transmission electron microscope (TEM). Dislocation loops with a size larger than 10 nm could be found in both W and W-3Re alloy, but the distribution of them was different. The distribution of the dislocation loops was more uniform in pure W, while they seemed to be clustered around some locations in W-3Re alloy. Voids (~2.4 nm) were observed in W-3Re alloy, while no void was investigated in W. High-dose irradiation induced obvious hardening with the hardening rate between 75% and 155% in all W-xRe alloys, but W-3Re alloy had the lowest hardening rate (75%). The main reasons might be related to the smallest grain size in W-3Re alloy, which suppressed the formation of defect clusters and induced smaller hardening than that in other samples.

## 1. Introduction

W and its alloys have been widely studied for their potential to be used as a candidate material for fusion reactor divertor (plasma-facing first wall) since they have superior characteristics (such as high melting point, low sputtering rate and low tritium retention, et al.) [1,2], compared with other nuclear materials (low activation steels [3], vanadium alloys [4], SiC/SiC composites [5], Zr-based alloys [6], etc.). In a fusion reactor, materials would be subjected to extreme conditions, such as high-energy, high-flux density neutron irradiation, high-concentration H/He plasma irradiation and high thermal load [7,8,9,10]. At the same time, transmutation elements (such as Re and Os elements) would be produced in the materials during neutron irradiation. As a result, various precipitated phases and a large number of defects would be induced in the W matrix during its service, which would lead to the hardening of materials and deterioration of properties, threatening the stable operation of fusion reactors [11,12,13]. Meanwhile, the Re content could be reached as high as 3.8 at.% in the W material after only 5 years of irradiation [14,15]. Thus, it is meaningful to study the evolution of micro defects and hardening behavior under irradiation in W and its alloys with different Re content in detail.

W-xRe alloys with different Re content were often used to simulate the effect of transmutation element Re on the properties of W and its alloy under neutron irradiation in a fusion reactor [16,17]. It was reported that pure W and W-Re alloys are usually fabricated by powder metallurgy and a laser powder melting method due to their high melting point [18,19]. Armstrong et al. found that the grain size of pure W using a powder metallurgy route could be affected by adding 5% Re, which changed from 50–500 μm (pure W) to 10–100 μm (W-5Re alloy) [18]. Yamamoto et al. [19] prepared W-Re alloys with different contents of Re (0, 1, 3, 10 wt.%) by the laser powder melting method. The result showed that the addition of Re content would not only influence their surface topography (such as grain and surface crack) but also affected the mechanical properties (such as hardening and softening at different temperatures) under an un-irradiation condition.

Neutron irradiation (low-dose, <10 dpa) and ion irradiation were widely used in simulation research for fusion neutron irradiation, as no real fusion neutron source exists at present [17,20]. Under neutron or ion irradiation, W-xRe alloys would induce a lower ductile-brittle transition temperature, smaller irradiated defect size and lower irradiated hardening than pure W [21,22,23]. Fukuda et al. [16]. found that Re could inhibit the formation of vacancies or dislocations in W and W-Re alloy under 1 dpa irradiation at 500 °C and 800 °C respectively. Hwang et al. used neutron irradiation (0.96 dpa at 538 °C and 0.90 dpa at 500 °C) combined with atom probe tomography (APT) analysis to show that the Re element was easily enriched at irradiation defects [24].

Compared with neutron irradiation, it is easier to obtain higher damage values by ion irradiation, which is necessary to predict the properties of materials in long-term service. Jiang et al. [25] observed the voids in the W when irradiated with 4 MeV W^2+^ up to 44 dpa at room temperature. Armstrong et al. and Xu et al. [18,20,26] used 2 MeV W^+^ to irradiate a tungsten alloy up to 33 dpa at different temperatures of 573 K and 773 K, and the agglomeration of alloying elements was the main influence on hardening, and it was close to saturation at ~13 dpa when considering the hardening due to dislocation loop damage in W and W-5Re alloy. So far, most studies had focused on radiation damage at doses less than 50 dpa; however, the lifetime damage of a fusion reactor was close to 150 dpa [27]. Therefore, the influence of Re in W-xRe alloys under a high dose (larger than 50 dpa) asks for a comprehensive understanding.

It was found that Si ions were advisable to be chosen for high-dose irradiation since the dislocation structures generated by Si ions irradiation and W ions irradiation (self-ion irradiation) were very similar in W material [28]. In this study, Si^2+^ irradiation of 2.7 MeV was performed in five kinds of W-xRe (x = 0, 1, 3, 5 and 10 wt.%) alloys at 550 °C and the corresponding peak damage was as high as 100 dpa. The surface topography change, irradiated defects, and irradiated hardening were studied using SEM, TEM and nano-indentation tests. The effect of Re content on grain size, dislocation loops, voids and irradiated hardening was investigated. This study provides a reference under high-dose radiation damage.

## 2. Materials and Methods

**Figure 1:**
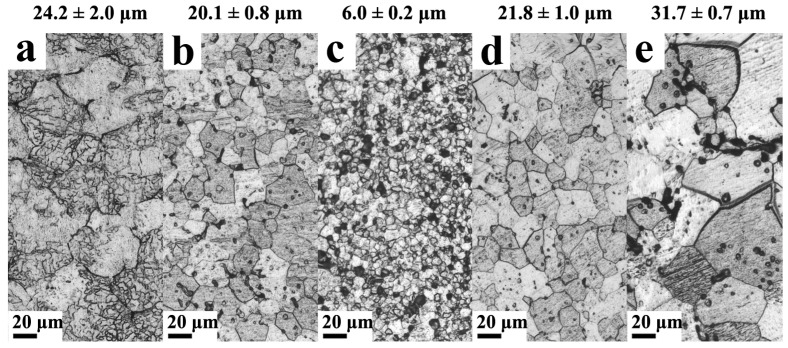
The grain size of samples with different Re contents: (**a**) W, (**b**) W-1Re alloy, (**c**) W-3Re alloy, (**d**) W-5Re alloy, (**e**) W-10Re alloy. The grain size of the samples was shown above the image.

The samples used in this experiment were provided by Hunan Rheniumet Rhenium Alloy Limited Company (Changsha, China). All samples (20 mm × 20 mm × 0.5 mm) were annealed at 1400 °C for 1 h in a hydrogen atmosphere annealing furnace. After annealing, the samples were cut into sheets of 2 mm × 3 mm × 0.5 mm. Silicon carbide (SIC) sandpaper with different mesh numbers (400#, 600#, 800#, 1000#, 2000# and 5000#) was used to polish successively to remove the surface oxide layer. Electrochemical polishing (the polishing solution was 0.4 mol/L NaOH aqueous solution, constant voltage mode and polishing voltage was 10 V, the polishing temperature was room temperature and polishing time was 20 s) was used to remove the fine scratches and strain layer generated by mechanical polishing. The grain size of the samples (3 pieces for each sample) was observed by a metallographic microscope, as shown in Figure 1 and summarized in Table 1.

The irradiation experiments were carried out on the 2 × 1.7 MV tandem accelerator (GIC4117, General Ionex, Ipswich, MA, USA) in the Accelerator Laboratory of Wuhan University [29]. The upgraded ion source (cesium-sputtering negative ion source) could provide high flux and stable Si^2+^ beams using solid Si palladium material. The irradiation area of the target chamber was 6 mm × 6 mm, and the ion source was about 10 m away from the target chamber. The temperature was controlled to 550 ± 5 °C throughout the irradiation, which was monitored by a thermocouple. The sample was irradiated to peak damage of 100 dpa for 80,240 s, and the vacuum degree was higher than 2.0 × 10^−4^ Pa in the whole irradiation process. 

**Figure 2:**
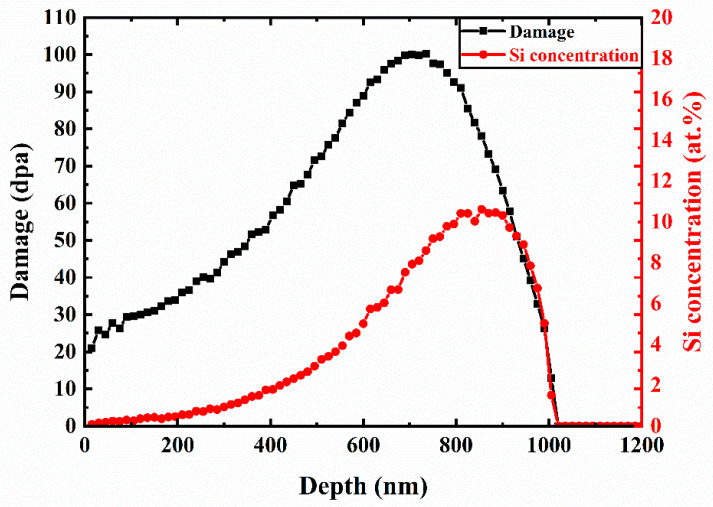
SRIM calculation of damage and concentration under 2.7 MeV Si^2+^ irradiation with a fluence of 3.06 × 10^21^ ions m^−2^ in pure W.

Figure 2 showed SRIM simulation calculation of 2.7 MeV Si^2+^ ion implantation into pure tungsten that reached peak damage of 100 dpa using the model of Ion Distribution and Quick Calculation of Damage [30], and displacement energy of 90 eV [31], a total of 100,000 ions simulation were performed. The dose rate at different depths could be simulated by dividing the dose by the total irradiation time, as a result the dose rate corresponding to the damage peak was 1.2 × 10^−3^ dpa/s.

The grain size and the surface morphology of samples before and after irradiation were performed with an optical microscope (RX50M, Sunny Optical Technology, Yuyao, China) and SEM (MIRA3, TESCAN, Brno, Czech Republic), equipped with a field-emission electron gun. All samples were photographed at an acceleration voltage of 20 KeV with 10,000 times magnification. W and W-3Re TEM sample with an appropriate thickness (~ 40 nm) were prepared using a focusing ion beam (FIB) device (Helios G4 UX dual-beam FIB, FEI, Waltham, MA, USA) and observed using a transmission electron microscopy (TEM) device (JEOL 2100, Tokyo, Japan, equipped with a B_6_La filament with an acceleration voltage of 200 KeV). The most often used image conditions were bright-filed images with g = 011 near the [−111] axis. For FIB preparation, a Pt film was deposited on the surface of the samples to protect the surface. After grooving and thinning to 2 μm using a 30 KeV Ga^+^ ion source, the samples was transferred to a pre-prepared copper mesh using a nano-manipulator. The ion beam energy was gradually reduced to further thin the samples to 40 nm. Finally, 2 KeV Ga^+^ was used to clean the samples’ surface to remove the damage caused by the high-energy ion beam on the samples’ surface. As a result, the size of the TEM samples was obtained to be 3 μm × 4 μm × 40 nm (thickness).

In order to compare the hardness of W-xRe alloys before and after high-dose irradiation with different Re content, all samples were measured in the nano-indentation instrument Nano Indenter G200* produced by Agilent Company in Wuhan University. The instrument was equipped with a Berkovich diamond indenter with a 20 nm radius of curvature at the tip of the indenter. The maximum load in the experiment was 500 mN, the loading mode was an electromagnetic force, the allowable drift rate was 0.05 nm/s, the frequency target was 45 Hz, the harmonic displacement target was 2 nm, and the percent to unload was 90%. Sixteen pressing points were randomly selected for each sample, and multiple continuous curves of hardness distribution with depth were obtained by using CSM mode (i.e., the continuous stiffness measurement mode). The depth between 0–1500 nm (completely including the irradiation area) was selected, and the strain rate was 0.05 s^−1^ by default. The distance between any two points was larger than 50 μm to avoid the influence of different points.

## 3. Results

### 3.1. Microstructure

#### 3.1.1. Surface Topography

**Figure 3:**
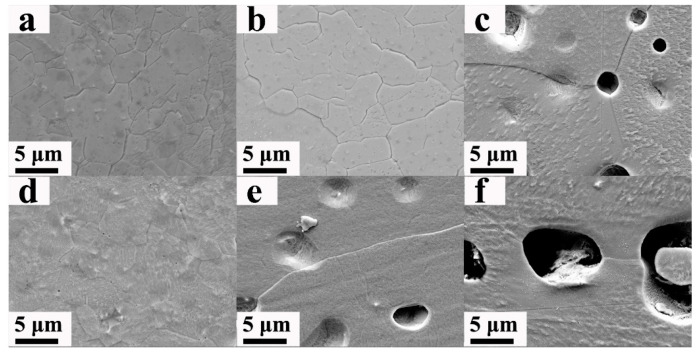
SEM images of samples before and after irradiation: (**a**) unirradiated W, (**b**) irradiated W, (**c**) irradiated W-1Re alloy, (**d**) irradiated W-3Re alloy, (**e**) irradiated W-5Re alloy, (**f**) irradiated W-10Re alloy.

Figure 3a showed the surface topography of W before irradiation, and Figure 3b–f corresponds to the surface topography of W-xRe alloys (x is 0, 1, 3, 5 and 10, respectively) after 100 dpa irradiation. No obvious change could be found on the surface of the samples before and after irradiation. However, the surface of the W alloy would form obvious blistering after it is irradiated with a high concentration of light mass ions (D^+^/He^+^), such as He ions with a fluence of 1.04 × 10^22^ m^−2^ at 60 KeV [32] or D plasma with a total fluence of 2.2 × 10^25^ m^−2^ [33]. W atoms could be knocked away from the equilibrium position to form a self-interstitial atom (SIA), implanted gas ions (He or D) in W would be trapped by the resulting vacancies, He-vacancies or D-vacancies clusters and other impurity atoms, and then formed bubbles. The bubbles agglomerated and form blisters. Thus, the aggregation of high-concentration gas ions was the main reason for the blistering, which was different from Si ion irradiation. As a result, no obvious change on the surface of the samples existed after irradiation in our work, though the total Si ion fluence reached 3.06 × 10^21^ m^−2^.

#### 3.1.2. Dislocation Loops

**Figure 4:**
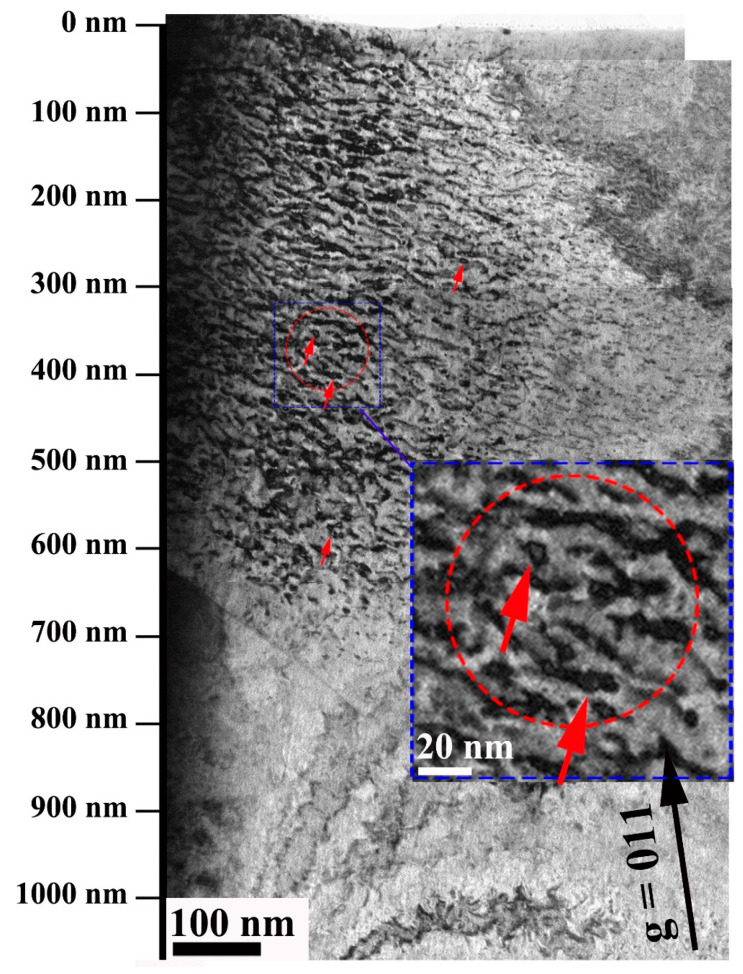
TEM image of dislocation loops distribution with depth in W sample, using g = 011 near the [−111] axis in the upper part. The arrows denote the typical dislocation loops, with color having no meaning.

Two grains with different crystal orientations could be observed in the upper and lower parts of Figure 4. In the upper part, obvious dislocation loops (marked by arrows) were observed using g = 011 near the [−111] axis in sample W after 100 dpa irradiation. The location of damage peaks was very close to the results of the SRIM simulation. Moreover, it could be found that small-size dislocation loops were closely intertwined with the large-size dislocation lines (marked by dotted circle). To avoid the surface effect in the surface area and high-ion concentration near the damage peak, the depth between 300–600 nm was selected for statistical analysis of the dislocation loops.

**Figure 5:**
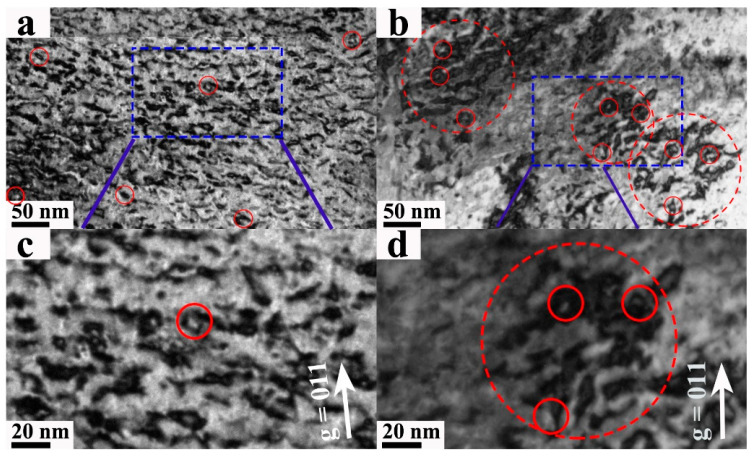
Images of the dislocation loops in the 300–600 nm range after 100 dpa irradiation using g = 011 near the [−111] axis: (**a**) W, (**b**) W-3Re alloy; (**c**,**d**) are higher magnification images of the corresponding regions marked with blue squares in (**a**,**b**). The solid circles denote the typical dislocation loops, with color having no meaning.

Figure 5 showed the images of the dislocation loops (red circle) of W and W-3Re alloy samples in the 300–600 nm region. Compared with W-3Re alloy, the distribution of the dislocation loops in pure W was more uniform, while the dislocation loops in W-3Re alloy seemed to be clustered around some locations. Several regions (500 nm × 300 nm × 40 nm) were chosen for each sample to analyze the distribution of the dislocation loops. The size and number density of the dislocation loops were 12.5 ± 3.8 nm and (1.90 ± 0.36) ×10^22^ m^−3^ in W, 11.6 ± 2.7 nm and (1.51 ± 0.25) × 10^22^ m^−3^ in W-3Re alloy, respectively, as shown in Table 1.

#### 3.1.3. Voids

**Figure 6:**
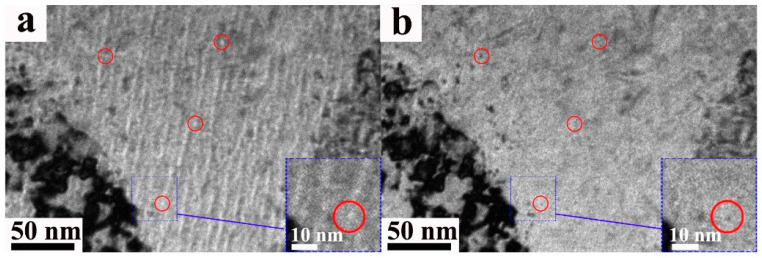
Images of the voids in W-3Re alloy after 100 dpa irradiation: (**a**) under-focus image, (**b**) over-focus image. The circles denote the typical voids, with color having no meaning.

No void was observed in all regions of the W sample. However, in the W-3Re alloy, small voids were observed in the depth range of 400 nm to 600 nm, as shown in Figure 6. The under-focus image and the over-focus image were shown in Figure 6a,b. Some of the typical voids were marked with red circles. Three regions (500 nm × 200 nm × 40 nm) were chosen to analyze the distribution of the voids. The mean size of the voids in the W-3Re alloy is 2.4 ± 0.6 nm, as summarized in Table 1.

### 3.2. Nano-Indentation Test

**Figure 7:**
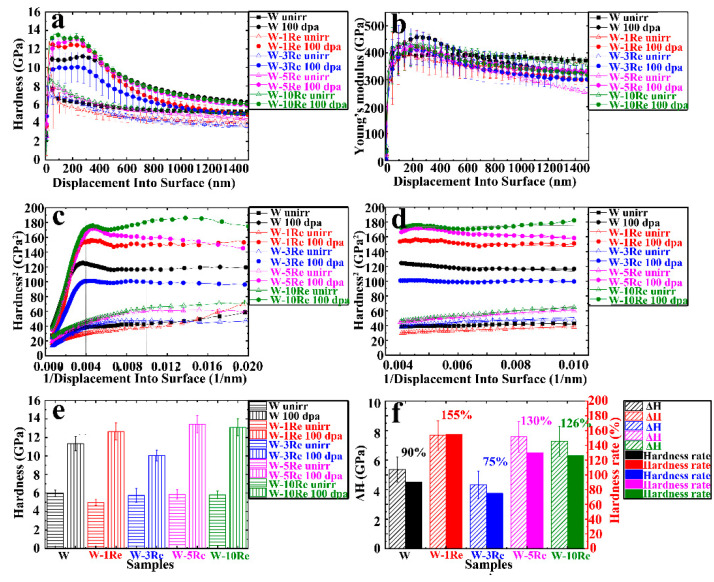
Nanoindentation test results: (**a**) average curve of hardness with depth, (**b**) average curve of Young’s modulus with depth, (**c**) by transforming (**a**) through the Nix-Gao model, (**d**) linear fitting of data in the range of 0.004–0.01 1/nm (corresponding to a depth range of 100 nm to 250 nm), (**e**) hardness value obtained by fitting, (**f**) ΔH and hardening rate of all 5 samples.

Figure 7a,b showed the curve of hardness and Young’s modulus with depth obtained by the nanoindentation test before and after the irradiation of all samples. A total of 1500 nm depth contained several areas that had different influences on hardness, including surface region (<50 nm, surface effect is obvious, such as surface oxidation layer and surface fluctuation), irradiation region (the influence of microscopic defects caused by irradiation is mainly on hardness) and soft substrate region (unirradiated area) [34]. Due to the existence of the indentation size effect (ISE) and soft substrate effect (SSE) [35], nanoindentation results were not strictly corresponding to the actual depth of samples. In order to obtain more accurate hardness values and remove the influence of ISE, the Nix-Gao model used for data fitting was as follows [36]:

(1) $$H^{2}=H_{0}{}^{2}1+\frac{h^{*}}{h}$$

where *H* (GPa) is the hardness measured at depth *h* (nm), *H*_0_ (GPa) is the matrix hardness at infinite depth, and h^*^ (nm) is the characteristic length only related to the indenter and material.

Figure 7a was transformed by Formula (1) to get the curve of H^2^ − 1/h, as shown in Figure 7c. On both sides where 1/h was 0.004 (i.e., depth of 250 nm), H had a completely different variation trend with 1/h, indicating that the influence of the substrate on hardness after 250 nm depth could not be ignored; the area close to the surface (0–100 nm) was abandoned and the depth range from 100 nm to 250 nm was selected for linear fitting. As shown in Figure 7d, the intercept of the fitting line was the square of the actual hardness value. Finally, the hardness obtained was shown in Figure 7e and summarized in Table 1.

As a result, the hardness values of W, W-1Re alloy, W-3Re alloy, W-5Re alloy and W-10Re alloy before irradiation were 5.97 ± 0.35 GPa, 4.97 ± 0.33 GPa, 5.74 ± 0.74 GPa, 5.85 ± 0.49 GPa and 5.80 ± 0.42 GPa, repectively. The hardness values changed little with the addition of Re element in W, since the hardness of all samples was less than 1 GPa and the error effect could not be ignored. After irradiation, the hardness gap of the samples became larger. The hardness values of W, W-1Re alloy, W-3Re alloy, W-5Re alloy and W-10Re alloy after irradiation were 11.33 ± 0.76 GPa, 12.65 ± 0.93 GPa, 10.06 ± 0.58 GPa, 13.43 ± 0.93 GPa and 13.09 ± 0.93 GPa, repectively. As a result, the hardness rate of W-3Re alloy was the smallest.

The hardening rate could better reflect the variation of hardness of different samples, and the specific definition was as follows:

(2) $$\mathrm{Harding}\;\mathrm{rate}\;\left(\%\right)=\frac{H^{irr}-H^{unirr}}{H^{unirr}}\times 100\%=\frac{\Delta H}{H^{unirr}}\times 100\%$$

where *H^irr^* is the hardness values of the samples after irradiation, *H^unirr^* is the hardness values of the samples before irradiation, and Δ*H* represents the difference value of *H^irr^* and *H^unirr^*. Δ*H* and hardening rate of all 5 samples were shown in Figure 7f. The irradiation hardening rates of W, W-1Re alloy, W-3Re alloy, W-5Re alloy and W-10Re alloy are 90%, 155%, 75%, 130% and 126%, respectively.

## 4. Discussion

### 4.1. Effect of Re Content on Grain Size

The addition of the Re element could significantly refine the grain, which was consistent in many studies [37,38]. Different from the smallest grain size of W-10Re alloy in Ref. [37], W-3Re alloy had a smallest grain size in our work, which might be due to the different fabrication. Similar to the existence of Mo-rich particles in W-Mo alloys [39], Ravi Kiran et al. also observed the formation of Re-rich particles in their prepared W-Re alloys [40]. It showed that Re had an important effect on the growth kinetics of W grain and was closely related to temperature.

Meanwhile, it was found that nanoporous material was likely to exhibit significant resistance to structural damage since it has a large concentration of sinks [41,42]. In our result, the grain sizes of the W, W-1Re alloy, W-3Re alloy, W-5Re alloy and W-10Re alloy were 24.2 ± 2.0 μm, 20.1 ± 0.8 μm, 6.0 ± 0.2 μm, 21.8 ± 1.0 μm and 31.7 ± 0.7 μm, respectively. The addition of 3% Re in pure W could significantly reduce the grain size, resulting in a large concentration of sinks that could remove point defects and the nucleation and growth of dislocation loops, which might reduce the irradiation damage.

### 4.2. Effect of Re Content on Microstructure

As one of the most common micro defects produced by irradiation, dislocation loops, are very important to the properties of materials. Yi et al. found that the evolution of dislocation loops has different laws with different doses in pure W and W-alloys using in-situ W^+^ irradiation [22]. When irradiated with the dose below 0.2 dpa, the nucleation and growth of dislocation loops were less affected by the surrounding dislocation loops, while the dose reached 0.4 dpa, dislocation loops began to gather together to form strings. As the dose was further increased, a lot of dislocation loops were interwoven to form a dislocation network. In our samples, few independent dislocation loops were observed, as most of them were intertwined with the dislocation lines. The possible reason is that the evolution of the dislocation loops may have stabilized earlier when irradiated under very high doses.

Even the low addition of Re element also could significantly reduce the remote mobility of SIA clusters in the W-xRe alloy, which was conducive to the merger of one-dimensional migration clusters, promoting the nucleation, aggregation and growth of loops [43]. The SIA clusters generated by irradiation had a certain probability of merging with the dislocation loops, but the influence on the dislocation loops density was small. Our results showed that the size and number density of the dislocation loops in W-3Re alloy were slightly smaller than those in W. Different from the distribution of the dislocation loops in W, the dislocation loops in W-3Re alloy had a regional agglomeration phenomenon, which might be attributable to the strong pinning effect of Re clusters on the dislocation loops under irradiation, limiting the movement of the dislocation loops.

Obvious voids were observed in W and its alloy under low neutron irradiation damage (about 1.5 dpa) with a relatively wide temperature range (400–800 °C), but the size of voids was generally less than 3 nm [17,44,45]. Ion irradiation showed that the lower dose rate (10^−4^ dpa/s) contributed to the formation of larger voids [19]. However, we did not observe the voids in pure W, but in W-3Re alloy. First principles calculations showed that the short-range order (SRO) parameter between Re and vacancy was negative, showing an attractive effect on vacancy [46]. Neutron irradiation experiments also showed that the voids could be decorated by Re and Os atoms [47,48]. Therefore, the aggregation of Re atoms and the formation of voids were mutually promoted under the irradiation of high-dose ions. In pure tungsten, due to the high dose rate (the dose rate corresponding to the damage peak is 1.2 × 10^−3^ dpa/s), the vacancy had no time to agglomerate into nucleation, while in W-3Re alloy, the cluster of Re elements promoted the vacancy to agglomerate into voids. In our work, small voids were observed in the W-3Re alloy with a depth from 400 nm to 600 nm, which might be related to the corresponding dose rate (according to SRIM simulation, the corresponding dose was 56.7–88.8 dpa at depth between 405–600 nm, dose rate was 7.1 × 10^−4^–1.1×10^−3^ dpa/s).

### 4.3. Effect of Re Content on Hardness under High-Dose Irradiation

The samples with different Re contents all showed obvious hardening after 100 dpa irradiation. Hwang et al. reported that the hardness value of W was 6.6 GPa and 9.3 GPa before and after 1dpa self-ion irradiation at 500 °C, and the hardening was saturated after irradiation to 1 dpa [23]. However, Xu et al. found that the hardness value of W-2Re alloy was 5.85 Gpa and 9.69 GPa before and after self-ion irradiation at 500 °C up to 33 dpa [26]. Similarly, Armstrong et al. also irradiated W and W-5Re alloy with 2 MeV self-ions at 300 °C to a maximum of 33 dpa, and the hardening rate reached 45% in W-5Re alloy [18]. Khan et al. irradiated W with ions of different energies at 400 °C to reach peak damage of 40 dpa and increased the hardness of annealed samples by 23% [49]. As the above results were obtained at different irradiation conditions, the irradiation hardening seemed to be saturated. However, the hardening rates in our work were between 75% and 155% in all samples after 100 dpa irradiation, which were much higher than the above experimental results, indicating that the hardness had not reached saturation with such low dose reported above.

The irradiation hardening rates of W, W-1Re alloy, W-3Re alloy, W-5Re alloy, and W-10Re alloy are 90%, 155%, 75%, 130%, and 126%, respectively. As a result, the addition of 3% Re element could the reduce the irradiation hardening in W, while the addition of 1%, 5% and 10% Re element would increase the irradiation hardening, which was consistent with the result of previous researches [18,23]. Hwang et al. found the irradiation hardening of W-3Re alloy was lower than W when irradiated to 5 dpa with self-ion at 500 °C and 800 °C [23]. It is reported that the hardness in W would be increased by adding 5% Re element [18]. The hardness increment was 2.88 Gpa in W-5Re alloy, which was higher than that in W (0.92 GPa).

It was well established that the defect morphology could be estimated by the change of hardness values (ΔH) using the dispersed barrier-hardening (DBH) mode [50]: ΔH ≈ 3.06 Δσ = 3.06 Mαμb$\sqrt{Nd}$, where Δσ (GPa) is yield strength change, M is the Taylor factor, α is the barrier strength factor, μ is the shear modulus, b (nm) is the Burgers vector, N is the number density of defect and d is the average size for the defect. The equation showed that the ΔH (GPa) would be proportional to the $\sqrt{Nd}$ value. In our work, the $\sqrt{Nd}$ values in W and W-3Re alloy after irradiation were calculated to be 0.015 1/nm and 0.013 1/nm, respectively. As a result, the ΔH (GPa) in W would be larger than that in W-3Re alloy, corresponding with the trend in our nanoindentation test. Only the contribution of dislocation loops was taken into account in both samples, since no voids were found in W and the average size of voids observed in W-3Re alloy was 2.4 nm; as a result, most of the voids (< 2 nm) could not contribute to the irradiation hardening [51].

At the same time, $\sqrt{Nd}$ value in W-xRe alloy could be estimated according to the $\sqrt{Nd}$ value (0.015 1/nm) in W and the measured ΔH by nanoindentation test, which could reflect the defect morphology after irradiation. The $\sqrt{Nd}$ value of the defects in W-1Re alloy, W-3Re alloy, W-5Re alloy and W-10Re alloy could be estimated to be 0.021 1/nm, 0.012 1/nm, 0.021 1/nm and 0.020 1/nm, respectively. For W-3Re alloy, the $\sqrt{Nd}$ value (0.012 1/nm) is close to that calculated by the dislocation loops observed in this work (0.013 1/nm). Compared with pure W, the higher $\sqrt{Nd}$ value might be contributed by higher density defects and the precipitates that formed in other three alloys after irradiation. It was reported that dislocation loop densities in the W-5Re alloy were higher than in pure W, but loops were smaller under the same irradiation conditions [16]. Further to the dislocation loops and voids, the contribution of precipitates on the hardening of W-Re alloy with high Re could not be neglected. Tanno et al. found that χ phase precipitates (Re_3_W) would be formed after neutron irradiated to 0.96 dpa at 538 °C, which were the main contribution to the large irradiation hardening of W-5Re alloy and W-10Re alloy [51].

In our work, the hardening rate was smallest in W-3Re alloy after irradiation, which might be related to the smallest grain size in it, suppressing the formation of defect clusters and inducing smaller irradiation hardening than that in other samples [41]. If the influence of ion types of irradiations was not taken into account, higher damage would lead to greater hardening, which was difficult to assess through the statistics of microscopic defects. Because the defects visible under TEM were only a part of the source of hardening, the invisible defects and the interaction between them could not be ignored, nanoindentation technology could give a good qualitative result.

## 5. Conclusions

The effect of Re content on microstructural changes and irradiated hardening in five kinds of W-xRe (x = 0, 1, 3, 5 and 10 wt.%) alloys under high-dose Si^2+^ irradiation up to 100 dpa at 550 °C were investigated. The main conclusions are as follows:

(1) The addition of the Re element could significantly refine the grain in the W-xRe alloys, and no obvious surface topography changed under high-dose Si ion irradiation.

(2) Dislocation loops were found in both W and W-3Re alloys, but their distribution of them was different. The distribution of dislocation loops was more uniform in pure W, while the dislocation loops seemed seem to be clustered around some locations in W-3Re alloy, which may be attributable to the strong pinning effect of Re clusters on the dislocation loops under irradiation, limiting the movement of the dislocation loops.

(3) Voids (~2.4 nm) were observed in W-3Re alloy, while no void was investigated in W, which showed that the cluster of Re elements might promote the vacancy to agglomerate into voids.

(4) Among the five Re contents investigated, W-3Re alloy exhibited the best performance in the suppression of the irradiation hardening. The main reasons might be related to the smallest grain size in W-3Re alloy, which suppressed the formation of defect clusters and induced smaller hardening than that in other samples.

## Figures and Tables

**Table 1:** Statistical data of samples before and after irradiation.

Sample	Grain Size (μm)	Dislocation Loop		Voids Size (nm)	Hardness	
		Loops Size (nm)	Number Density (×10^22^ m^−3^)		Un-Irradiation (GPa)	Irradiation (GPa)
W	24.2 ± 2.0	12.5 ± 3.8	1.90 ± 0.36	------	5.97 ± 0.35	11.33± 0.76
W-1Re	20.1 ± 0.8	------	------	------	4.97 ± 0.33	12.65 ± 0.93
W-3Re	6.0 ± 0.2	11.6 ± 2.7	1.51 ± 0.25	2.4 ± 0.6	5.74 ± 0.74	10.06 ± 0.58
W-5Re	21.8 ± 1.0	------	------	------	5.85 ± 0.49	13.43 ± 0.93
W-10Re	31.7 ± 0.7	------	------	------	5.80 ± 0.42	13.09 ± 0.93

## Data Availability

The data presented in this study are available on request from the corresponding author.

## References

[1] Barrett T.R., Ellwood G., Pérez G., Kovari M., Fursdon M., Domptail F., Kirk S., McIntosh S.C., Roberts S., Zheng S. (2016). Progress in the Engineering Design and Assessment of the European DEMO First Wall and Divertor Plasma Facing Components. Fusion Eng. Des..

[2] You J.H., Visca E., Barrett T., Böswirth B., Crescenzi F., Domptail F., Fursdon M., Gallay F., Ghidersa B.E., Greuner H. (2018). European Divertor Target Concepts for DEMO: Design Rationales and High Heat Flux Performance. Nucl. Mater. Energy.

[3] Qiu G., Zhan D., Li C., Qi M., Yang Y., Jiang Z., Zhang H. (2019). Effects of Yttrium on Microstructure Stability and Tensile Properties of China Low Activation Martensitic Steel. Metals.

[4] Muroga T., Chen J.M., Chernov V.M., Kurtz R.J., Le Flem M. (2014). Present Status of Vanadium Alloys for Fusion Applications. J. Nucl. Mater..

[5] Katoh Y., Snead L.L., Henager C.H., Nozawa T., Hinoki T., Iveković A., Novak S., Gonzalez De Vicente S.M. (2014). Current Status and Recent Research Achievements in SiC/SiC Composites. J. Nucl. Mater..

[6] Kashkarov E., Nikitenkov N., Sutygina A., Laptev R., Bordulev Y., Obrosov A., Liedke M.O., Wagner A., Zak A., Weiβ S. (2018). Microstructure, Defect Structure and Hydrogen Trapping in Zirconium Alloy Zr-1Nb Treated by Plasma Immersion Ti Ion Implantation and Deposition. J. Alloys Compd..

[7] Liu D.G., Zheng L., Luo L.M., Zan X., Song J.P., Xu Q., Zhu X.Y., Wu Y.C. (2018). An Overview of Oxidation-Resistant Tungsten Alloys for Nuclear Fusion. J. Alloys Compd..

[8] Lin J.S., Hao Y.C., Luo L.M., Zhao M.L., Xu Q., Zan X., Zhu X.Y., Wu Y.C. (2018). Microstructure and Performances of W–TiC–Y2O3 Composites Prepared by Mechano-Chemical and Wet-Chemical Methods. J. Alloys Compd..

[9] Pitts R.A., Carpentier S., Escourbiac F., Hirai T., Komarov V., Kukushkin A.S., Lisgo S., Loarte A., Merola M., Mitteau R. (2011). Physics Basis and Design of the ITER Plasma-Facing Components. J. Nucl. Mater..

[10] Yu P.F., Pan B.C. (2022). Performance of Tungsten Nitride Compound Surfaces to Resist Sputtering under Intense Irradiation in Nuclear Fusion Reactors. Appl. Surf. Sci..

[11] Tanno T., Hasegawa A., Fujiwara M., He J.C., Nogami S., Satou M., Shishido T., Abe K. (2008). Precipitation of Solid Transmutation Elements in Irradiated Tungsten Alloys. Mater. Trans..

[12] Cottrell G.A., Pampin R., Taylor N.P. (2006). Transmutation and Phase Stability of Tungsten Armor in Fusion Power Plants. Fusion Sci. Technol..

[13] Terentyev D., Yin C., Dubinko A., Chang C.C., You J.H. (2021). Neutron Irradiation Hardening across ITER Diverter Tungsten Armor. Int. J. Refract. Met. Hard Mater..

[14] Gilbert M.R., Sublet J.C. (2011). Neutron-Induced Transmutation Effects in W and W-Alloys in a Fusion Environment. Nucl. Fusion.

[15] Knaster J., Moeslang A., Muroga T. (2016). Materials Research for Fusion. Nat. Phys..

[16] Fukuda M., Yabuuchi K., Nogami S., Hasegawa A., Tanaka T. (2014). Microstructural Development of Tungsten and Tungsten-Rhenium Alloys Due to Neutron Irradiation in HFIR. J. Nucl. Mater..

[17] Hasegawa A., Fukuda M., Nogami S., Yabuuchi K. (2014). Neutron Irradiation Effects on Tungsten Materials. Fusion Eng. Des..

[18] Armstrong D.E.J., Yi X., Marquis E.A., Roberts S.G. (2013). Hardening of Self Ion Implanted Tungsten and Tungsten 5-Wt% Rhenium. J. Nucl. Mater..

[19] Yamamoto T., Hara M., Hatano Y. (2021). Cracking Behavior and Microstructural, Mechanical and Thermal Characteristics of Tungsten–Rhenium Binary Alloys Fabricated by Laser Powder Bed Fusion. Int. J. Refract. Met. Hard Mater..

[20] Xu A., Armstrong D.E.J., Beck C., Moody M.P., Smith G.D.W., Bagot P.A.J., Roberts S.G. (2017). Ion-Irradiation Induced Clustering in W-Re-Ta, W-Re and W-Ta Alloys: An Atom Probe Tomography and Nanoindentation Study. Acta Mater..

[21] Nogami S., Terentyev D., Zinovev A., Yin C., Rieth M., Pintsuk G., Hasegawa A. (2021). Neutron Irradiation Tolerance of Potassium-Doped and Rhenium-Alloyed Tungsten. J. Nucl. Mater..

[22] Yi X., Jenkins M.L., Kirk M.A., Zhou Z., Roberts S.G. (2016). In-Situ TEM Studies of 150 KeV W+ Ion Irradiated W and W-Alloys: Damage Production and Microstructural Evolution. Acta Mater..

[23] Hwang T., Fukuda M., Nogami S., Hasegawa A., Usami H., Yabuuchi K., Ozawa K., Tanigawa H. (2016). Effect of Self-Ion Irradiation on Hardening and Microstructure of Tungsten. Nucl. Mater. Energy.

[24] Hwang T., Hasegawa A., Tomura K., Ebisawa N., Toyama T., Nagai Y., Fukuda M., Miyazawa T., Tanaka T., Nogami S. (2018). Effect of Neutron Irradiation on Rhenium Cluster Formation in Tungsten and Tungsten-Rhenium Alloys. J. Nucl. Mater..

[25] Jiang W., Zhu Y., Zhang L., Edwards D.J., Overman N.R., Nandipati G., Setyawan W., Henager C.H., Kurtz R.J. (2021). Dose Rate Effects on Damage Accumulation and Void Growth in Self-Ion Irradiated Tungsten. J. Nucl. Mater..

[26] Xu A., Beck C., Armstrong D.E.J., Rajan K., Smith G.D.W., Bagot P.A.J., Roberts S.G. (2015). Ion-Irradiation-Induced Clustering in W-Re and W-Re-Os Alloys: A Comparative Study Using Atom Probe Tomography and Nanoindentation Measurements. Acta Mater..

[27] Kemp R., Cottrell G.A., Bhadeshia H.K.D.H. (2007). Designing Optimised Experiments for the International Fusion Materials Irradiation Facility. J. Nucl. Mater..

[28] Shao L., Wei C.C., Gigax J., Aitkaliyeva A., Chen D., Sencer B.H., Garner F.A. (2014). Effect of Defect Imbalance on Void Swelling Distributions Produced in Pure Iron Irradiated with 3.5 MeV Self-Ions. J. Nucl. Mater..

[29] Chen Y., Guo L., Long Y., Xie Z., Luo H., Lin W. (2022). Nuclear Inst. and Methods in Physics Research, A Establishment of Multi-Beam Irradiation Facility at Wuhan University. Nucl. Inst. Methods Phys. Res. A.

[30] Luo H., Luo F., Chen Y., Wang J., Liu Q., Li F., Xie Z., Lin W., Guo L. (2022). Effect of Yttrium Content on Microstructure and Irradiation Behavior of V-4Cr-4Ti-XY Alloys. J. Nucl. Mater..

[31] Sharma P., Maya P., Akkireddy S., Raole P.M., Tyagi A.K., Attri A., Kulriya P.K., Bajpai P.K., Mishra S., Patel S.P. (2019). Effect of Heavy Mass Ion (Gold) and Light Mass Ion (Boron) Irradiation on Microstructure of Tungsten. Microsc. Microanal..

[32] Zhang R., Zhou H., Yu J., Han W., Liu M., Chen C., Zhu K. (2018). Effect of Vanadium Alloying on Irradiation Performance of Tungsten under 60 keV Helium Irradiation. J. Nucl. Mater..

[33] Wang Z., Gao L., Zhu X.L., Yuan Y., Wang S., Cheng L., Lu G.H. (2021). Effect of Rhenium on Defects Evolution Behavior in Tungsten under Irradiation. Nucl. Fusion.

[34] Jiang S.N., Xu L.Q., Zheng P.F. (2018). Evaluation of Hardening Behavior under Synergistic Interaction of He and Subsequent H Ions Irradiation in Vanadium Alloys. Nucl. Mater. Energy.

[35] Kasada R., Takayama Y., Yabuuchi K., Kimura A. (2011). A New Approach to Evaluate Irradiation Hardening of Ion-Irradiated Ferritic Alloys by Nano-Indentation Techniques. Fusion Eng. Des..

[36] Nix W.D., Gao H. (1998). Indentation Size Effects in Crystalline Materials: A Law for Strain Gradient Plasticity. J. Mech. Phys. Solids.

[37] Kappacher J., Leitner A., Kiener D., Clemens H., Maier-Kiener V. (2020). Thermally Activated Deformation Mechanisms and Solid Solution Softening in W-Re Alloys Investigated via High Temperature Nanoindentation. Mater. Des..

[38] Wang Y.M., Tang Q.H., Zhou P. (2021). Microstructure and Magnetron Sputtering Properties of W/Re Alloy Targets Fabricated by Vacuum Sintering. J. Mater. Eng. Perform..

[39] Hsu C.S., Lin S.T. (2002). Coalescence of Tungsten Grains around Molybdenum Grains in the Presence of a Liquid Phase. Scr. Mater..

[40] Ravi Kiran U., Panchal A., Prem Kumar M., Sankaranarayana M., Nageswara Rao G.V.S., Nandy T.K. (2017). Refractory Metal Alloying: A New Method for Improving Mechanical Properties of Tungsten Heavy Alloys. J. Alloys Compd..

[41] Aradi E., Lewis-Fell J., Harrison R.W., Greaves G., Mir A.H., Donnelly S.E., Hinks J.A. (2018). Enhanced Radiation Tolerance of Tungsten Nanoparticles to He Ion Irradiation. Nanomaterials.

[42] Bringa E.M., Monk J.D., Caro A., Misra A., Zepeda-Ruiz L., Duchaineau M., Abraham F., Nastasi M., Picraux S.T., Wang Y.Q. (2012). Are Nanoporous Materials Radiation Resistant?. Nano Lett..

[43] Castin N., Bonny G., Bakaev A., Ortiz C.J., Sand A.E., Terentyev D. (2018). Object Kinetic Monte Carlo Model for Neutron and Ion Irradiation in Tungsten: Impact of Transmutation and Carbon Impurities. J. Nucl. Mater..

[44] Van Renterghem W., Bonny G., Terentyev D. (2022). TEM Investigation of Neutron Irradiated and Post Irradiation Annealed Tungsten Materials. Fusion Eng. Des..

[45] Fukuda M., Hasegawa A., Nogami S., Yabuuchi K. (2014). Microstructure Development of Dispersion-Strengthened Tungsten Due to Neutron Irradiation. J. Nucl. Mater..

[46] Nguyen-Manh D., Wróbel J.S., Klimenkov M., Lloyd M.J., Messina L., Dudarev S.L. (2021). First-Principles Model for Voids Decorated by Transmutation Solutes: Short-Range Order Effects and Application to Neutron Irradiated Tungsten. Phys. Rev. Mater..

[47] Lloyd M.J., Abernethy R.G., Gilbert M.R., Griffiths I., Bagot P.A.J., Nguyen-Manh D., Moody M.P., Armstrong D.E.J. (2019). Decoration of Voids with Rhenium and Osmium Transmutation Products in Neutron Irradiated Single Crystal Tungsten. Scr. Mater..

[48] Lloyd M.J., London A.J., Haley J.C., Gilbert M.R., Becquart C.S., Domain C., Martinez E., Moody M.P., Bagot P.A.J., Nguyen-Manh D. (2022). Interaction of Transmutation Products with Precipitates, Dislocations and Grain Boundaries in Neutron Irradiated W. Materialia.

[49] Khan A., Elliman R., Corr C., Lim J.J.H., Forrest A., Mummery P., Evans L.M. (2016). Effect of Rhenium Irradiations on the Mechanical Properties of Tungsten for Nuclear Fusion Applications. J. Nucl. Mater..

[50] El-Atwani O., Weaver J.S., Esquivel E., Efe M., Chancey M.R., Wang Y.Q., Maloy S.A., Mara N. (2018). Nanohardness Measurements of Heavy Ion Irradiated Coarse- and Nanocrystalline-Grained Tungsten at Room and High Temperature. J. Nucl. Mater..

[51] Tanno T., Fukuda M., Nogami S., Hasegawa A. (2011). Microstructure Development in Neutron Irradiated Tungsten Alloys. Mater. Trans..

